# Optimizing Structural and Mechanical Properties of an Industrial Ti-6246 Alloy below β-Transus Transition Temperature through Thermomechanical Processing

**DOI:** 10.3390/ma17051145

**Published:** 2024-03-01

**Authors:** Mohammed Hayder Ismail Alluaibi, Irina Varvara Balkan, Nicolae Șerban, Ion Cinca, Mariana Lucia Angelescu, Elisabeta Mirela Cojocaru, Saleh Sabah Alturaihi, Vasile Dănuț Cojocaru

**Affiliations:** 1 Materials Science and Engineering Faculty, University Politehnica of Bucharest, 060042 Bucharest, Romania; mohammed.alluaibi@kus.edu.iq (M.H.I.A.); nicolae.serban@upb.ro (N.Ș.); ion.cinca@upb.ro (I.C.); mariana.angelescu@upb.ro (M.L.A.); elisabeta.cojocaru@upb.ro (E.M.C.); dan.cojocaru@upb.ro (V.D.C.); 2 Department of Medical Physics, College of Science, Al-Karkh University of Science, Baghdad 10081, Iraq; 3 Department of Metallurgical Engineering, Faculty of Materials Engineering, University of Babylon, Al Hillah 51002, Iraq; eng486.salah.sabah@uobabylon.edu.iq

**Keywords:** titanium alloys, hot deformation, solution treatment, microstructure, mechanical properties

## Abstract

This study aims to investigate the effect of hot deformation on commercially available Ti-6246 alloy below its β-transus transition temperature at 900 °C, knowing that the α → β transition temperature of Ti-6246 alloy is about 935 °C. The study systematically applies a thermomechanical processing cycle, including hot rolling at 900 °C and solution and ageing treatments at various temperatures, to investigate microstructural and mechanical alterations. The solution treatments are performed at temperatures of 800 °C, 900 °C and 1000 °C, i.e., below and above the β-transus transition temperature, for 9 min, followed by oil quenching. The ageing treatment is performed at 600 °C for 6 h, followed by air quenching. Employing various techniques, such as X-ray diffraction, scanning electron microscopy, optical microscopy, tensile strength and microhardness testing, the research identifies crucial changes in the alloy’s constituent phases and morphology during thermomechanical processing. In solution treatment conditions, it was found that at temperatures of 800 °C and 900 °C, the α′-Ti martensite phase was generated in the primary α-Ti phase according to Burger’s relation, but the recrystallization process was preferred at a temperature of 900 °C, while at a temperature of 1000 °C, the α″-Ti martensite phase was generated in the primary β-Ti phase according to Burger’s relation. The ageing treatment conditions cause the α′-Ti/α″-Ti martensite phases to revert to their α-Ti/β-Ti primary phases. The mechanical properties, in terms of strength and ductility, underwent an important beneficial evolution when applying solution treatment, followed by ageing treatment, which provided an optimal mixture of strength and ductility. This paper provides engineers with the opportunity to understand the mechanical performance of Ti-6246 alloy under applied stresses and to improve its applications by designing highly efficient components, particularly military engine components, ultimately contributing to advances in technology and materials science.

## 1. Introduction

Titanium and its alloys are particularly important materials for modern technology due to their superior mechanical characteristics, especially at high temperatures and their relatively low specific weight (γ = 4.51 gf/cm^3^). Thus, the specific weight of titanium occupies a central place between the specific weights of the two main metals used in industry, iron (7.86) and aluminum (2.70), respectively.

Being positioned in seventh place among the most abundant metals (aluminum, iron, magnesium, etc.) in the earth’s crust, with a proportion of almost 0.6%, titanium has raised interest in many industrial applications, such as aerospace, automotive and medical devices, due to its excellent characteristics. Prominent properties that are routinely evaluated and given special consideration include titanium’s notable resistance to erosion and corrosion across various media, coupled with its low density and high strength. Additionally, it exhibits moderate strength at elevated temperatures, commendable biocompatibility and reasonable resistance to oxidation. Setting it apart from other lightweight metals, titanium distinguishes itself by its intricate physical metallurgy and unique characteristics [1].

Titanium and its alloys are often used instead of steels due to their higher melting point, rendering them valuable as refractory materials. However, the practical temperature limits for CP-Ti in structural applications range from less than 427 °C (800 °F) to approximately 538 °C or 595 °C (1000 °F or 1100 °F), contingent on the specific chemical composition of titanium-based alloys. Distinctive properties, including good conductivity, efficient thermal transfer and a favorable thermal expansion coefficient, have garnered attention for titanium’s application in diverse industries, such as electronics, automotive and aerospace [2,3,4].

When classifying titanium-based alloys, we can recognize three important groups, namely: titanium alloys of α-type, (α + β)-type and β-type. The α-type and β-type classes are also split into two subclasses, namely: titanium alloys of near-α-Ti-type and near-β-Ti-type. The α allotropic state is stable at low temperatures in the form of a compact hexagonal lattice, while the β allotropic state is stable at high temperatures and possesses a volume-centered cubic lattice.

In principle, the large grain structure of a metal undergoing allotropic transformations can be improved by heat treatment. The heat treatment of metallic titanium can be performed by heating it to a temperature above the allotropic transformation point and cooling it at different rates. Thus, multiple studies have delved into exploring the impact of mechanical working and heat treatment on the microstructures of α and near-α alloys [5,6,7,8], α + β alloys [9,10,11,12,13], near-β and β alloys [14,15,16,17,18,19,20,21,22]. For the in-depth examination of titanium-based alloys and the consequences of their thermomechanical processing on microstructure evolution and mechanical properties, detailed reviews are available [23,24,25,26,27,28,29,30].

Ti-6Al-2Sn-4Zr-6Mo (Ti-6246) is a titanium alloy developed by Pratt and Whitney in 1966, with a focus on high-strength applications, particularly in military engines like the F-100 and F-119. It performs well at temperatures between 315 °C and 400 °C and has a tensile strength of 1170 MPa, a yield strength of 1035 MPa and an elongation of 10% or higher. In addition to its military engine applications, the Ti-6246 alloy composition includes various alloying elements to achieve its desired properties. The specific balance of components in the alloy contributes to its high strength and thermal stability. Although its use is widespread in military aviation, its limitations in damage tolerance compared to Ti-6Al-4V and Ti-6Al-2Sn-4Zr-2Mo alloys make it less preferable for commercial engines, where extended inspection intervals are crucial for operational efficiency and safety. The alloy’s development reflects the continuous pursuit of optimal materials for specific engineering requirements in aerospace applications [23,31,32].

The utilization of Ti-6246 alloy in gas turbine engines extends to critical components, such as compressor disks and fan blades, where its high-strength characteristics are advantageous in intermediate-temperature sections. The alloy’s versatility also includes its use in seals and various airframe components, contributing to the overall performance and reliability of the engine. Beyond aerospace, ongoing assessments of deep sour-well applications highlight its potential to challenge environments beyond traditional turbine settings, emphasizing its adaptability and the continuous exploration of its capabilities in diverse industrial contexts [23,31].

In the field of metallurgy and materials engineering, the optimization of structural and mechanical properties is paramount for enhancing the performance of industrial alloys. This intricate process involves a nuanced interplay in thermomechanical processing parameters. It is a meticulous symphony, orchestrating deformation, cooling and temperature and duration treatments to induce desirable changes in Ti-6246 alloy’s crystal structure, which are strategically applied to manipulate the alloy’s microstructure and mechanical behavior. By navigating the alloy’s phasic transformations below the β-transus temperature, researchers and engineers endeavor to tailor its properties, ensuring optimal strength and ductility. This systematic approach not only improves mechanical behavior but also extends the improvement journey beyond mechanical properties, seeks to mitigate potential challenges, such as thermal stresses and microstructural inhomogeneities, and delves into thermal stability and overall material reliability, ensuring a robust alloy with consistent and predictable performance characteristics. The researchers are exploring the alloy’s response to thermomechanical processing, aiming to achieve an ideal balance of properties that not only enhances its immediate performance but also opens up prospects for its use under different operating conditions and critical industrial environments, from aerospace components to structural applications requiring reliability and longevity. This multidisciplinary effort brings together metallurgists, materials scientists and engineers, uniting their expertise to unleash the full potential of the Ti-6246 alloy through meticulous thermomechanical processing below the β-transus temperature. The result is a finely tuned precision alloy that can meet the stringent requirements of modern industrial applications, where flexibility, efficiency and durability are paramount.

## 2. Materials and Methods

**Figure 1:**
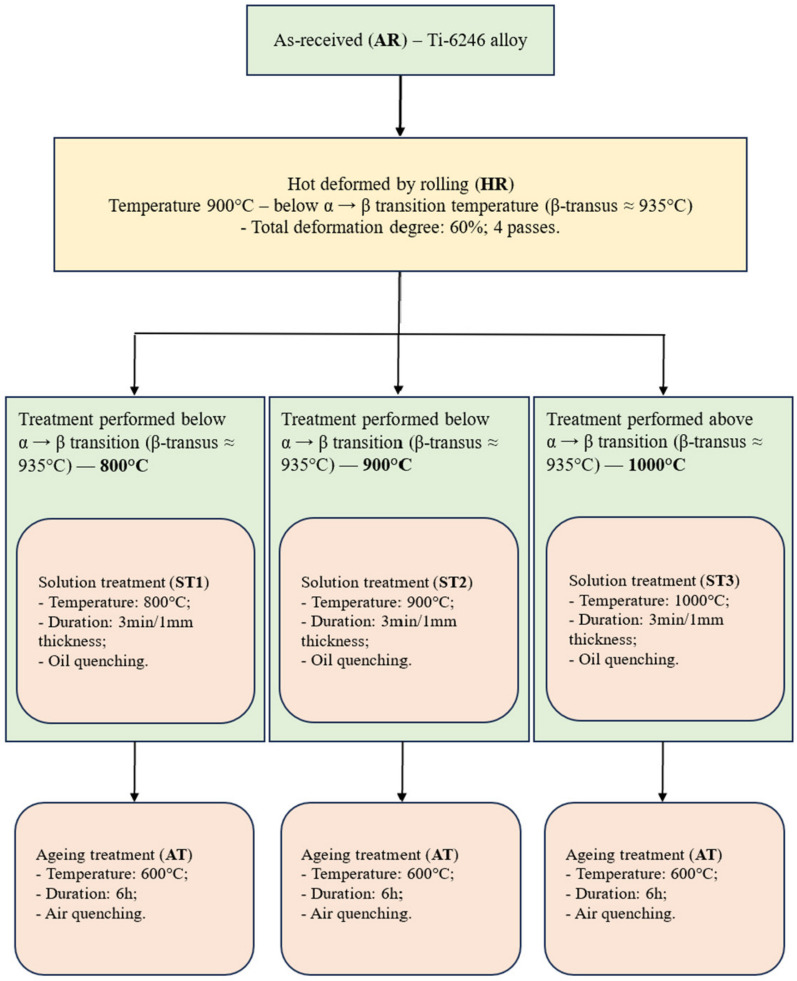
Thermomechanical processing route.

Based on relevant research experimental programs [1,33,34,35], Ti-6Al-2Sn-4Zr-6Mo (Ti-6246) alloy was investigated by thermomechanical (TM) processing in order to determine the optimal processing route to improve its microstructure and mechanical properties. The as-received (AR) Ti-6246 alloy, with a diameter of Ø600 mm, was industrially obtained from S.C. ZIROM TITANIUM S.A. in Giurgiu, Romania, utilizing a double vacuum arc remelting processing route. The samples were cut from the AR alloy to be thermomechanically processed by hot rolling (HR) at 900 °C and obtaining a deformation degree (total thickness reduction) of approx. 60% ($\varepsilon_{total}$ ≈ 60%), i.e., a fixed deformation degree of $\varepsilon_{partial}$ ≈ 15% through 4 rolling steps with reheating at each rolling step. After the hot-deformation process, thermal processing (solution and ageing treatments) was applied at three different temperature stages: (1) below α → β transition temperature—800 °C (ST1-state), (2) close to α → β transition temperature—900 °C (ST2-state) and (3) above α → β transition temperature—1000 °C (ST3-state). In all TM-processing stages of the alloy, a SiC rod heating furnace (NABERTHERM HT 08/16) was used. Figure 1 shows the thermomechanical processing steps that Ti-6246 alloy went through.

In solution treatment, according to the sample thickness of 3 mm, the processing time is 3 min for a sample thickness of 1 mm (i.e., 9 min for a sample thickness of 3 mm). The processed samples were soaked in oil as an oil quenching (OQ) step, aiming to induce α′-Ti and α″-Ti secondary phases to form within Ti-6246 alloy. To achieve an optimal harmony of mechanical properties, with a specific focus on reducing the weight fraction of α′-Ti and α″-Ti secondary phases and enhancing both strength and ductility, an ageing treatment (AT) is recommended. In the context of this study, the prescribed ageing treatment parameters involve exposure to 600 °C for 6 h with air quenching (AQ), aiming to instigate phasic transformations (α′-Ti → α-Ti and α″-Ti → β-Ti) as well as other phenomena, such as stress-relieving and reordering/restructuring in the main phases.

After undergoing TM processing, an in-depth exploration of the structural and mechanical characteristics of Ti-6246 alloy samples demanded meticulous metallographic preparation. Initially, the samples were subjected to hot mounting within a Ø30 mm cylindrical sampler, containing conductive phenolic powder, maintained at 150 °C for 7 min. The produced samples underwent a comprehensive preparation regimen, involving polishing with a 6 μm and 1 μm polycrystalline diamond suspension (180 s/step) after being ground in 6 steps using 180 to 1200-grit SiC paper (60 s/step). Vibro-polishing, using 0.02 μm colloidal silica combined with a 1/5 ratio of 20% H_2_O_2_ (3.6 ks), was followed by super-polishing as a final step with a 0.05 μm suspension of colloidal silica combined with a 1/5 ratio of 20% H_2_O_2_ (300 s/step). These comprehensive procedures were undertaken to ensure meticulous preparation, setting the stage for a precise examination of the structural and mechanical characteristics of Ti-6246 alloy samples.

In microstructure analysis, the changes that occurred in the AR Ti-6246 alloy in terms of constituent phases and their morphology after TM processing were followed by optical microscopy (OM), scanning electron microscopy (SEM) and X-ray diffraction (XRD) techniques. OM analysis used a Metkon IMM 901 inverted optical microscope (Metkon Instruments, Bursa, Turkey). SEM analysis used a TESCAN Vega II-XMU (TESCAN, Brno, Czech Republic). It also used a Bruker Quantax 6/30 xFlash (Bruker Corporation, Billerica, MA, USA) detector to detect the alloy’s chemical composition computationally. XRD measurements used a Malvern Panalytical Empirean (Malvern Panalytical BV, Almelo, The Netherlands) diffractometer.

In mechanical analysis, all TM-processed samples were used for tensile and microhardness testing, which was performed at room temperature to verify the key mechanical parameters, namely: (1) ultimate tensile strength (σ_UTS_), (2) 0.2% yield strength (σ_0.2%_), (3) fracture elongation (ε_f_) and (4) microhardness (HV). For the tensile test, the samples were machined into “dog-bone-like” samples, which were calibrated at 50 × 5 × 1.5 mm and a crosshead speed of 2 mm/s. These rigorous evaluation testing procedures employed cutting-edge equipment, namely INSTRON 3382 universal testing equipment (INSTRON, Norwood, MA, USA) for tensile measurements and INNOVATEST Falcon 500 equipment (INNOVATEST Europe BV, Maastricht, Netherlands) for Vickers microhardness measurements. Mechanical properties were derived by averaging the values obtained from three tests performed on each TM-processed sample under identical conditions. The resultant mechanical properties were subjected to meticulous computation, including standard deviation. The mechanical properties were approximated as follows: (1) strength properties to the closest integer in MPa, (2) fracture elongation to 0.5% and (3) microhardness to 0.1 HV1.

## 3. Results and Discussion

### 3.1. Structural Development

**Figure 2:**
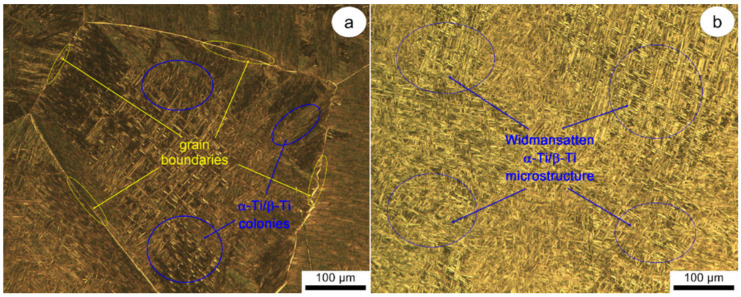
OM images of AR states across various investigation regions (**a**,**b**).

OM images of Ti-6246 alloy in its AR state are shown in Figure 2. Figure 2a shows that the microstructure contains a small portion of sub-millimeter-sized grains with an average grain size close to 500 μm but is composed primarily of millimeter-sized grains with an average grain size of 2 mm. In accordance with Widmanstätten/basket-weave type morphology, all grains exhibit the existence of several colonies with various spatial orientations, which are created by alternating fine lamellae/plate-like structures of the main phases (see Figure 2b).

**Figure 3:**
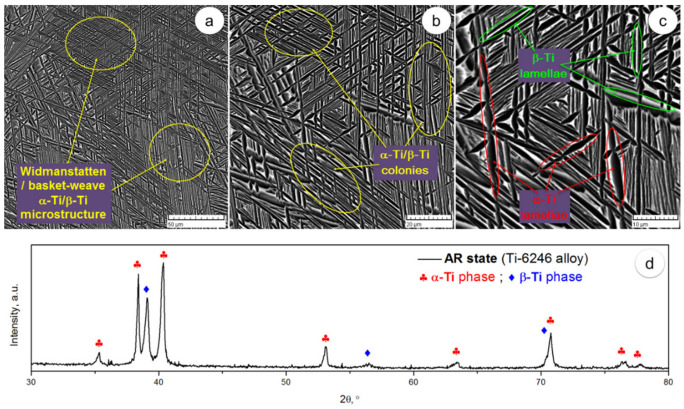
SEM-BSE images of AR state across various magnifications (**a**–**c**); XRD spectra of AR state (**d**).

Figure 3 shows SEM-back-scattered electrons (SEM-BSE) images of AR alloy. The microstructure shows alternating colonies of identically oriented lamellae/plate-like structures close to each other (see Figure 3a,b). It is possible to see the following constituent phases: (1) a phase with a light gray color, i.e., elemental components have a high atomic number and (2) a phase with a dark gray color, i.e., elemental components have a low atomic number (see Figure 3c). In addition, in both phases, the average lamella/platelet thickness was less than 1 μm. According to XRD analysis (Figure 3d), α-Ti and β-Ti phases were also observed. The crystalline structure of these phases is as follows: (1) The hexagonal close-packed lattice represents α-Ti phase, with lattice parameters near a = 0.294 nm and c = 0.467 nm; and (2) the volume-centered cubic lattice represents β-Ti phase, with lattice parameters near a = 0.327 nm. Table 1 shows the computed chemical composition of AR alloy.

**Figure 4:**
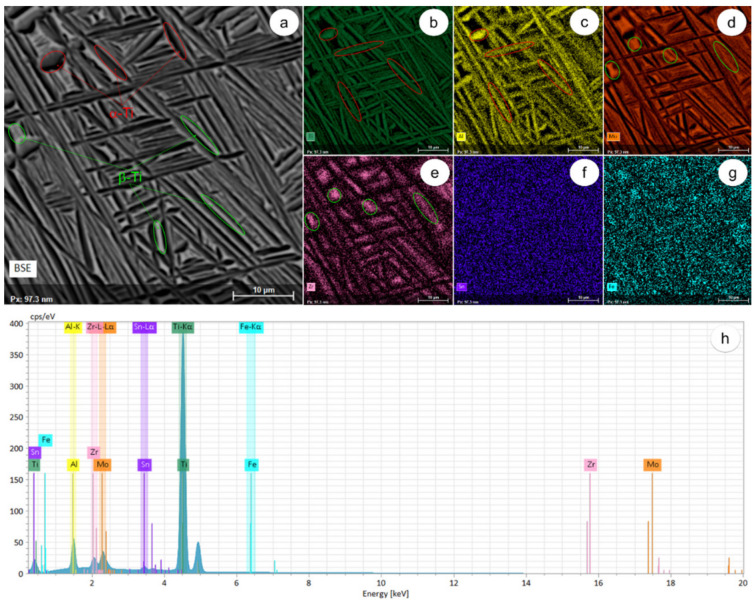
Distribution maps of elemental components in AR state: Characterized by SEM-BSE microstructural image (**a**); SEM-EDS distribution map of Ti (**b**); Al (**c**); Mo (**d**); Zr (**e**); Sn (**f**); Fe (**g**); global EDS spectra (**h**).

Figure 4 shows SEM-BSE and SEM-EDS images of AR alloy. Figure 4a shows a homogenous microstructure with no segregation between different phases and grains of micron size. Each grain comprises numerous cohesive colonies with alternating lamellae/plate-like structures of α-Ti and β-Ti phases, devoid of defects such as porosities, inclusions, etc. Figure 4b–g show the dispersion of the elemental components in the two main phases of Ti-6246 alloy. The α-Ti phase shows enrichment in α-stabilizer elements, specifically Ti and Al (Figure 4b,c), while being depleted in β-stabilizer elements, such as Mo and Zr (Figure 4d,e). Conversely, the β-Ti phase shows enrichment in β-stabilizer elements, specifically Mo and Zr (Figure 4d,e), and depletion in α-stabilizer elements, such as Ti and Al (Figure 4b,c). Sn and Fe (Figure 4f,g) have a moderate effect on the microstructure. Figure 4h shows the computed chemical composition of AR alloy by EDS spectral analysis, as detailed in Table 1. According to EDS analysis, the α-Ti phase is discerned by its dark gray color due to its enrichment in Ti and Al. Conversely, the β-Ti phase is discerned by its light gray color due to its enrichment in Mo and Zr.

**Figure 5:**
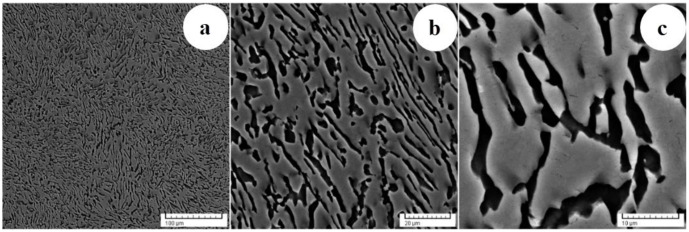
SEM-BSE images of HR state at 900 °C at various magnifications (**a**–**c**).

Figure 5 shows SEM-BSE images with different magnifications of HR state at 900 °C. The magnitude of deformation in the primary grains and grain boundaries is less (Figure 5a) when compared to the relevant study [33], which conducted the deformation temperature at 1000 °C, i.e., above the α → β transition temperature. This is because the deformation temperature is close to the α → β transition temperature, and, despite the high deformation applied in this state, the adjacent α-Ti/β-Ti colonies show less intense deformation, with elongation morphology tending to align along the rolling direction; they also have, on average, higher thickness (Figure 5b). The adjacent α-Ti/β-Ti colonies’ structures exhibit various morphologies and spatial orientations (Figure 5c). No thermally induced martensitic phases were observed in this state.

**Figure 6:**
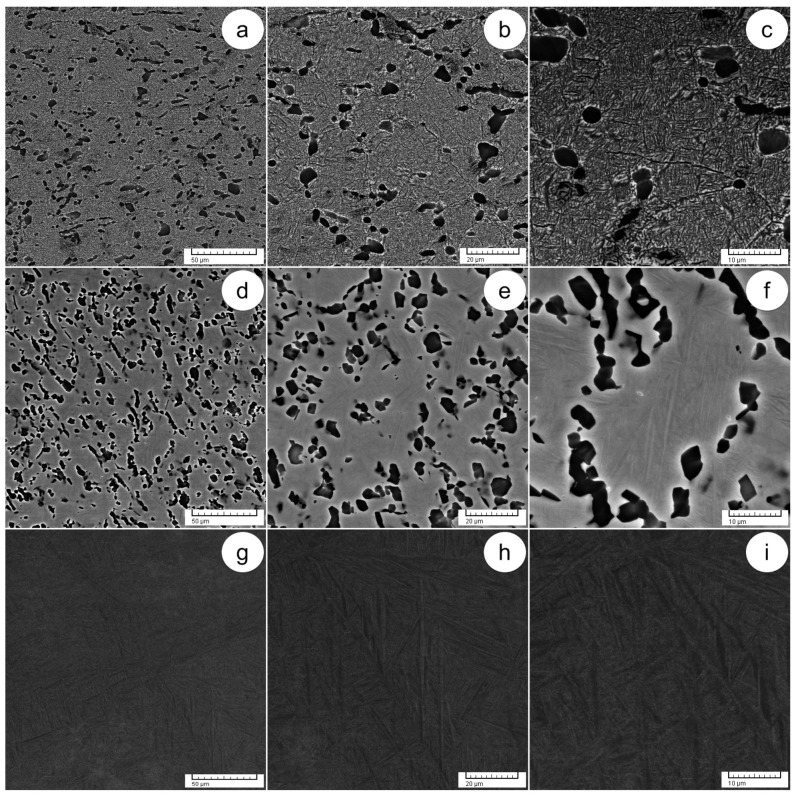
SEM-BSE images of solution treatments, including ST1 at 800 °C for 9 min. with OQ at various magnifications (**a**–**c**); ST2 at 900 °C for 9 min. with OQ (ST2) at various magnifications (**d**–**f**); ST3 at 1000 °C for 9 min. with OQ (ST3) at various magnifications (**g**–**i**).

Figure 6 shows the states of solution treatment at different temperatures and equal processing durations of 9 min (ST1—800 °C, 9 min. and OQ; ST2—900 °C, 9 min. and OQ; and ST3—1000 °C, 9 min. and OQ). In the state of ST1 (Figure 6a–c), meticulous observation shows that the deformed grains have been fully recrystallized, with each grain including alternating thin α-Ti/β-Ti lamellae/plate-like structures, indicating favored spatial orientation. In addition, rapid cooling to room temperature results in the generation of the α′-Ti phase, characterized by an acicular structure with a hexagonal close-packed lattice. Notably, the α′-Ti phase exhibits a favored spatial orientation with the primary α-Ti phase, aligning with the special Burger’s relation between the α-Ti and α′-Ti phases and sharing the same crystal lattice.

In the state of ST2 (Figure 6d–f), the microstructure shows a spotted coarse morphology of the primary α-Ti phase, larger than that of the ST1 state, as well as the lamellae/plate-like structures of the primary α-Ti and β-Ti phases. This leads to the observation that the recrystallization process in the α-Ti phase is much higher than in the β-Ti phase, indicating that heating to the ST2 state favors the recrystallization of the α-Ti phase. The martensitic α′-Ti phase, characterized by a platelet-like morphology and produced by rapid cooling to ambient temperature, can be seen within the primary α-Ti lamellae.

In the ST3 state (Figure 6g–i), the microstructure exhibits a distinctive basket-weave-like morphology characterized by a combination of lamellar/fine acicular structures and parallel platelets of large size. Notably, the secondary α″-Ti phase, featuring a parallel platelet structure with an orthorhombic crystal lattice, was also visible in this state in the primary β-Ti phase. This secondary phase is produced as an outcome of rapid cooling from 1000 °C to room temperature. Aligned with the special Burger’s relation between the primary β-Ti phase and the martensite α″-Ti phase, the martensite α″-Ti phase exhibits a favored spatial orientation with the primary β-Ti phase.

**Figure 7:**
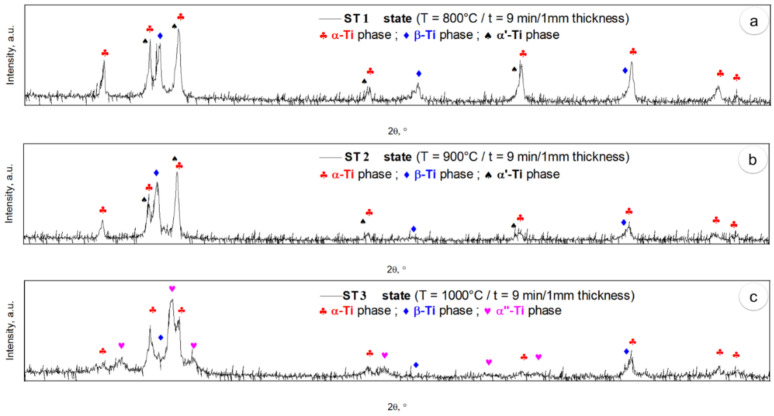
XRD spectra of solution treatments, including ST1 at 800 °C (**a**); ST2 at 900 °C (**b**); ST3 at 1000 °C (**c**).

Figure 7 presents XRD spectral plots for the different states of solution treatment. In both states, ST1 and ST2 (Figure 7a,b), distinct diffraction peaks of α-Ti, β-Ti and α′-Ti phases can be seen. Distinguishing between α-Ti and α′-Ti phases proves challenging due to their classification as hexagonal close-packed lattices with closely aligned lattice parameters (a = 0.294 nm and c = 0.467 nm). The subtle dissimilarities in lattice parameters result from the supersaturation of β-stabilizing elements in the martensitic α′-Ti phase compared to the primary α-Ti phase. In the state of ST3 (Figure 7c), distinct diffraction peaks of α-Ti, β-Ti and α″-Ti phases can be seen. The α″-Ti phase, characterized by an orthorhombic crystal lattice with lattice parameters close to a = 0.296 nm, b = 0.496 nm and c = 0.468 nm, is identified and is attributed to rapid cooling from 1000 °C to room temperature. Analysis of the relative intensities of the diffraction peaks shows a tendency toward phase texturing along specific crystallographic orientations, indicating favored crystallographic grain growth across all phases. When analyzing the width of the diffraction peaks, it can be indicated that there is a small grain size for all phases in all states, and this aligns with the SEM–BSE microstructural observations.

**Figure 8:**
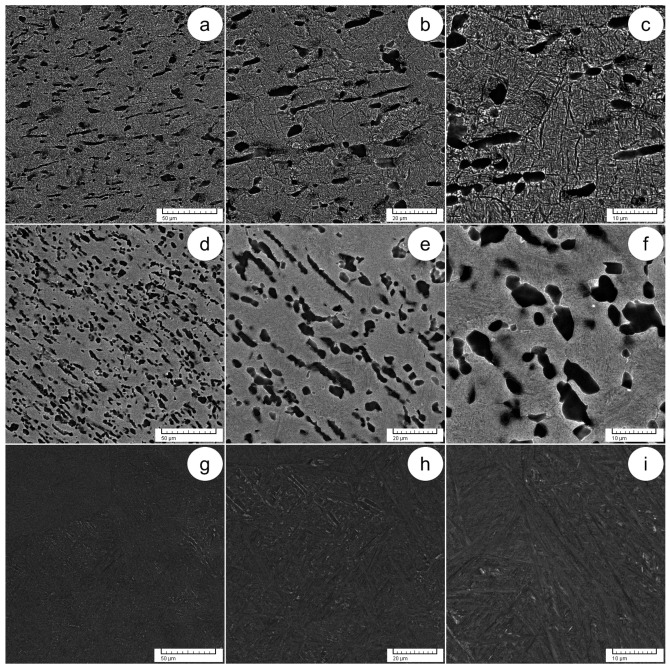
SEM-BSE images of ageing treatments, including ST1 + AT at 600 °C for 6 h. with AQ at various magnifications (**a**–**c**); ST2 + AT at various magnifications (**d**–**f**); ST3 + AT at various magnifications (**g**–**i**).

Figure 8 shows the states of ageing treatment under equal processing conditions after solution treatment (ST1 + AT—800 °C + 600 °C, 6 h and AQ, ST2 + AT—900 °C + 600 °C, 6 h and AQ and ST3 + AT—1000 °C + 600 °C, 6 h and AQ). In the states of ageing treatment, the occurrence of stress-relieving and phase transition α′-Ti/α″-Ti → α-Ti/β-Ti phenomena can be assumed. In the state of ageing treatment ST1 + AT (Figure 8a–c), the same morphological concept can be observed in terms of alternating α-Ti/β-Ti lamellae/plate-like structures, exhibiting favored spatial orientation. The spotted morphology of the α-Ti phase is also evident. After applying the ageing treatment, no fine acicular texture is visible, which may indicate that the treatment conditions at 600 °C, 6 h and AQ may be adequate to transform the α′-Ti phase into the primary α-Ti phase.

In the state of ageing treatment ST2 + AT (Figure 8d–f), the microstructure shows delicately sized α-Ti/β-Ti lamellae/plate-like structures as well as spotted morphologies. Due to the ageing treatment, which was adequate to return the α′-Ti phase to the primary α-Ti phase based on Burger’s relation between these phases, no α′-Ti phase was found in the microstructure.

In the state of ageing treatment ST3 + AT (Figure 8g–i), the microstructure exhibits a basket-weave-like morphology formed by the alternate fine lamellar/plate-like structures of the α-Ti and β-Ti phases. Due to the ageing treatment conditions that affected the phasic transformations α″-Ti → β-Ti, the presence of the α″-Ti phase was not observed.

The ageing process generally depends on the age-hardening curve, which describes the relationship between mechanical properties and the alloy’s ageing time at different ageing temperatures. The primary purpose of ageing treatment is to enhance the decomposition of secondary phases resulting from solution treatment. Therefore, selecting the ageing temperature and time is crucial to achieving the best performance in terms of the alloy’s overall properties. According to the literature review, the ageing temperature for titanium alloys of α + β type is around 500–600 °C, with a duration range of 4–12 h [36]. In contrast, for heat-treatable titanium alloys of β type, the ageing temperature is lower and the duration is longer, approximately 450 °C to 500 °C for 8 to 24 h [37]. Air cooling is commonly used for quenching.

### 3.2. Mechanical Properties Development

The mechanical properties 0.2% yield strength (σ_0.2%_), ultimate tensile strength (σ_UTS_), fracture elongation (ε_f_) and microhardness (HV1) are used to follow evolutions in the mechanical behavior of TM-processed samples. Table 2 lists the mechanical properties that were measured during the tensile and microhardness tests.

Comparison of the effects of hot deformation with the AR standard state (see Table 2) makes it obvious that the hot deformation causes a rise in strength and a drop in ductility (see Table 2). The observed behavior can be related to deformation-induced effects on the material’s microstructure. The initial grains, characterized by multiple cohesive colonies of alternating α-Ti/β-Ti lamellae/plate-like structures, are of less deformation compared to the relevant study [33] due to the hot deformation being applied under the β-transus transition temperature at 900 °C, reducing the increase in the microstructure defects density and the strain-hardening phenomenon. After hot deformation, the microstructure shows elongated morphology along the rolling direction. The microhardness development exhibits a rise in comparison to the AR standard state (see Table 2), which is attributed to the strain-hardening effects.

Important evolutions in the mechanical properties within the microstructure have been recorded in the states of solution treatment (ST1, ST2 and ST3). Sufficient treatment conditions below β-transus temperature cause the microstructure to recrystallize, producing an increase in strength and ductility properties and a decrease in defect density. According to the Hall–Petch relation, the strength increases as the grain size decreases. i.e., the mechanical properties are strongly influenced by the grain size (Equation (1)) [33], and this is consistent with what is observed in the microstructure shown in Figure 6 in the presence of (α-Ti/β-Ti/α′-Ti/α″-Ti) phases with low grain size.

(1) $$\sigma_{y}=\sigma_{0}+\frac{k}{\sqrt{d}}$$

where: σ_y_—yield strength; σ_0_—starting stress for dislocation movement; k—grain boundary resistance; d—grain size average.

It is essential to highlight that the phase with a higher weight fraction exerts a more pronounced influence on the material’s properties. In states of solution treatment, the microstructure consists of two main phases, namely α-Ti and β-Ti, along with other thermally induced phases, such as the martensite α′-Ti and α″-Ti phases. The α′-Ti phase appears when the solution is treated below the β-transus transition temperature as an outcome of rapid cooling and poses challenges in identification due to lattice parameters that match the primary α-Ti phase closely. Conversely, no transition occurs in the β-Ti phase (see Figure 7). The α″-Ti phase appears when the solution is treated above the β-transus transition temperature as an outcome of rapid cooling. Its identification is more straightforward, as it is thermally induced from the primary β-Ti phase, characterized by distinct crystal lattice and lattice parameters (see Figure 7).

The mechanical behavior of Ti-6246 alloy shows that the primary α-Ti and β-Ti phases are associated with raised strength and ductility properties, respectively. In addition, in comparison to the primary α-Ti and β-Ti phases along with their corresponding volumetric fractions, the behavior of the martensite α′-Ti and α″-Ti phases shows a combination of reduced strength and ductility properties. Based on these observations, it can be stated that ST is far below the β-transus transition temperature in which only α-Ti recrystallization and β-Ti phase restructuring occur. The α′-Ti phase produced by these treatment conditions causes an increase in mechanical properties. An ST close to the β-transus transition temperature also produces the α′-Ti phase but with a larger weight fraction, which implies a greater increase in the mechanical properties. In solution treatment above the β-transus temperature, the microstructure is of β-Ti phase only, and this state produces an α″-Ti phase with a large weight fraction, which causes a drop in strength and a rise in ductility.

In the ageing treatment states (ST1 + AT and ST2 + AT), compared to the solution treatment states carried out below the β-transus temperature, the observed behavior is a drop in strength and a rise in ductility, and this can be explained by the ageing treatment conditions being sufficient to cause the transition α′-Ti → α-Ti, which contributed to the drop in strength and rise in ductility. The microhardness showed a large increase due to the effect of ageing treatment, which may have served to precipitate very fine nanometer-sized strengthening particles of the α-Ti/ω-Ti phase. On the other hand, in the state of ageing treatment (ST3 + AT), compared to the solution treatment state carried out above the β-transus temperature, the ductility decreased and the strength increased due to ageing treatment conditions that were sufficient to cause the transition α″-Ti → α-Ti, and because of the rise in the weight fraction of the ω-Ti phase, resulting in a greater rise in the microhardness. When examining the weight fraction of the β-Ti phase in the states of ST1 + AT, ST2 + AT and ST3 + AT, a discernible trend emerges, indicating a rise in the weight fraction of the β-Ti phase with an increment in the ST temperature. Consequently, this increment affects the corresponding rise in the weight fraction of the transitioned α-Ti/ω-Ti phase during AT, ultimately contributing to the rise of the microhardness property.

Although the ω-Ti phase was not observed in the microstructure, it is believed that the precipitation of the ω-Ti phase in the α-Ti/β-Ti phases has occurred. The ageing duration required for the onset of the ω-Ti phase to assist in precipitation in the α-Ti phase is 4–8 h at a low ageing temperature, as in this paper, which used a titanium alloy of α + β type (aged at 600 °C for 6 h). Therefore, the focus will be on the ω-Ti phase in the second stage of studying the Ti-6246 alloy in addition to the martensitic phases [38,39].

In solution treatment, the relative quantity of phases depends on the solution treatment temperatures, which were favored in the process of recrystallization of the α-Ti phase in the case of ST2, as previously indicated. Other reasons can be pointed out in cases where high strength is achieved, one being the close spacing between incoherent particles, which hinders dislocation movement effectively, according to the Orowan relation, unlike in a lower-strength case, where the large spacing between incoherent particles facilitates effective dislocation movement. Additionally, from a crystallographic point of view, parallel colonies allow for easy slip transition, resulting in decreased strength properties. The secondary alpha phase does not significantly contribute to alloy hardening [12,40].

In ageing treatment, the transformation of the secondary α″-Ti phase and β-Ti phase into a fine needle-like structure leads to significant hardening of the material, where the hardness is linearly proportional to the amount of these fine phases [12,40].

**Figure 9:**
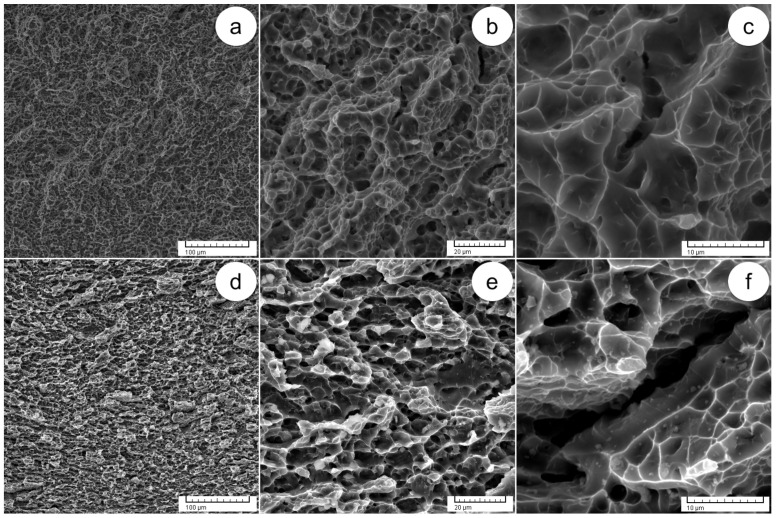
SEM–SE images of fracture surfaces following tensile testing: AR standard state at various magnifications (**a**–**c**); HR at 900 °C at various magnifications (**d**–**f**).

After the tensile test, the fracture surfaces were examined to observe the effect of thermomechanical processing on the tested samples. Figure 9 shows SEM–SE images of the fracture surfaces of both AR alloy and HR states. The fracture surfaces of the AR alloy show spongy morphologies, high density of voids and dimples and the presence of a void coalescence mechanism, demonstrating a clear ductility (Figure 9a–c). The ductile nature of the AR alloy supports the mechanical findings.

In the HR state (Figure 9d–f), distinct features include large crevices/fissures, cleavage surfaces and spongy regions. Within these spongy regions, smaller voids and shallow dimples are visible, exhibiting a lower density in comparison to the AR state. The boundaries of the deformed prior α-Ti/β-Ti colonies are represented by regions where void coalescence occurs. The mechanical findings align with these observations, confirming the presence of mixed brittle–ductile behavior in the HR state.

**Figure 10:**
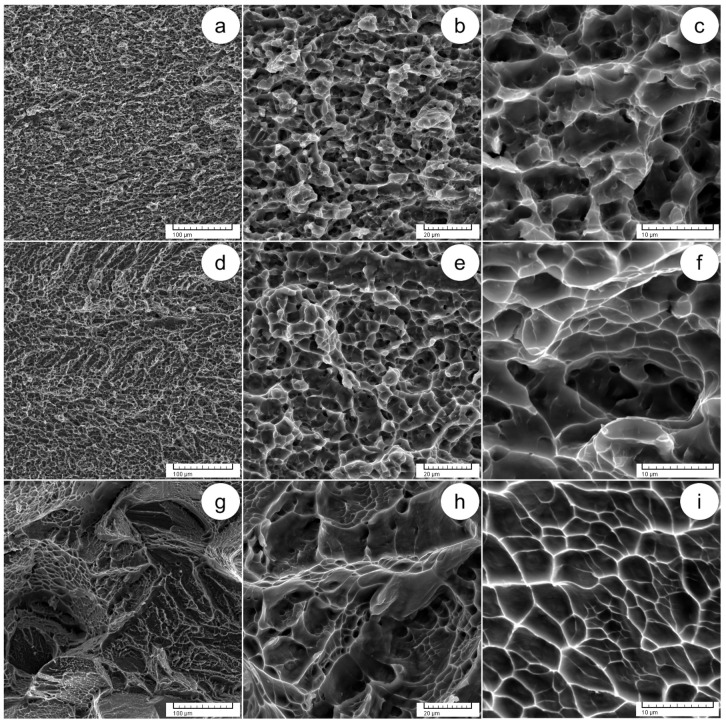
SEM–SE images of fracture surfaces following tensile testing, including ST1 at various magnifications (**a**–**c**); ST2 at various magnifications (**d**–**f**); ST3 at various magnifications (**g**–**i**).

Figure 10 shows SEM–SE images that provide detailed insight into the fracture surfaces across different ST states. Within the state of ST1 treatment (Figure 10a–c), observable features include small crevices/fissures neighboring spongy regions containing small-sized voids and shallow dimples. These observations suggest limited ductility and a nuanced interplay between brittle and ductile behaviors. Advancing to the state of ST2 (Figure 10d–f), the morphological observations bear a close resemblance to the state of ST1, in addition to being characterized by larger cleavage surfaces and a decrease in the density of spongy regions. The state of ST3 (Figure 10g–i) shows pronounced brittle behavior and moderate ductility, supporting the mechanical findings.

**Figure 11:**
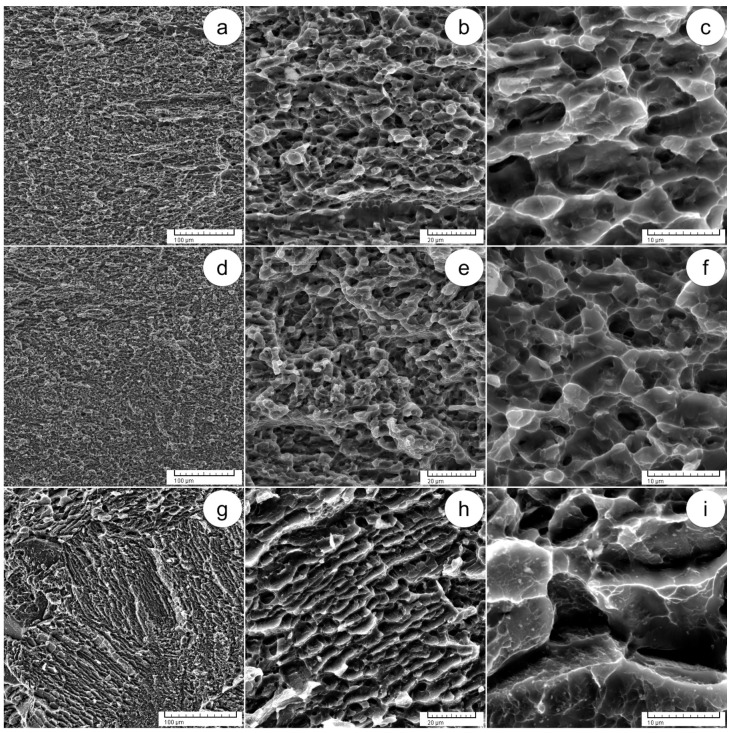
SEM–SE images of fracture surfaces following tensile testing, including ST1 + AT at various magnifications (**a**–**c**); ST2 + AT at various magnifications (**d**–**f**); ST3 + AT at various magnifications (**g**–**i**).

Figure 11 shows SEM–SE images that provide detailed insight into the fracture surfaces across different AT states. In the state of ST1 + AT, morphological observations reveal cleavage surfaces and small-sized crevices/fissures neighboring spongy regions, featuring small voids and shallow dimples (Figure 11a–c). This pattern indicates limited ductility, consistent with the mechanical findings, confirming the mixed coexistence of brittle and ductile behavior in the ST1 + AT state. Within the treatment state of ST2 + AT (Figure 11d–f), the examination of fracture surfaces reveals cleavage surfaces characterized by lower density in comparison to both the ST2 and ST1 + AT states. These smaller cleavage surfaces are situated near spongy regions, which are characterized by shallow dimples and small-sized voids, exhibiting a higher density than that observed in the ST2 and ST1 + AT states. Based on these morphological observations, the ST2 + AT state demonstrates high ductility, corroborating the mechanical findings. In the state of ST3 + AT (Figure 11g–i), the analysis of the fracture surface reveals small cleavage surfaces compared to the state of ST3, and the occurrence of spongy regions is minimized in comparison to the states of ST1 + AT and ST2 + AT. Small voids, shallow dimples and void coalescence regions are observed in the spongy regions, leading to decreased ductility and increased strength in comparison to the state of ST3. These morphological observations are consistent with the mechanical findings, proving the low ductility and high strength in the state of ST3 + AT.

## 4. Conclusions

After investigating the effects of hot deformation, followed by solution and ageing treatments, on Ti-6246 alloy, the following conclusions can be reached:

- The generation of the secondary α phase (α′-Ti), which affects the mechanical behavior of Ti-6246 alloy through improving both strength and ductility properties in comparison to the state of hot deformation, is induced by a solution treatment temperature that is maintained below the threshold of the β-transus transition temperature. It should be noted that the solution treatment temperature, which is performed close to the threshold of the β-transus transition temperature, achieves a high strength property.
- The generation of the secondary α phase (α″-Ti), which affects the mechanical behavior of Ti-6246 alloy through reducing strength and increasing ductility, is induced by the solution treatment temperature, which is performed above the threshold of the β-transus transition temperature.
- As a result of stress relief, dispersion-precipitation-strengthening mechanisms and the phase transitions α′-Ti/α″-Ti → α-Ti/β-Ti and β-Ti → α-Ti/ω-Ti, which occurred during ageing treatment, it is possible to nuancedly control the mechanical behavior of Ti-6246 alloy. This approach allows for the achievement of an optimal synergy between strength, ductility and microhardness properties.

Future studies will be about further improvements of Ti-6246 alloy by other TM processing routes in order to obtain mechanical properties with a balanced combination of higher quality.

## Figures and Tables

**Table 1:** Details of the elemental components of AR state.

Elemental Components	%, wt.	%, at	Abs. Error [%]	Rel. Error [%]
Ti	81.54	82.55	2.44	2.76
Al	6.33	11.36	0.33	4.77
Sn	1.85	0.75	0.05	2.94
Zr	3.91	2.08	0.16	3.79
Mo	6.24	3.15	0.24	3.54
Fe	0.14	0.12	0.12	2.77

**Table 2:** Mechanical properties during TM processing.

Structural Issue	Mechanical Properties			
	Microhardness, HV1	Ultimate Tensile Strength, σ_UTS_ [MPa]	0.2% Yield Strength, σ_0.2%_ [MPa]	Fracture Elongation, ε_f_ [%]
AR standard state	305.2 ± 16.9	1057 ± 14	967 ± 11	12.9 ± 1.8
HR	399.8 ± 13.7	1154 ± 14	996 ± 13	3.8 ± 0.8
ST1	373.5 ± 5.4	1165 ± 13	1008 ± 12	5.3 ± 1.1
ST2	301.8 ± 5.8	1357 ± 14	1174 ± 12	6.9 ± 1.2
ST3	320.4 ± 5.9	984 ± 13	785 ± 13	9.7 ± 1.2
ST1 + AT	397.8 ± 9.1	1089 ± 11	966 ± 13	8.1 ± 1.3
ST2 + AT	428.1 ± 5.3	1284 ± 12	1101 ± 14	15.4 ± 1.2
ST3 + AT	442.6 ± 3.7	1170 ± 11	986 ± 13	6.9 ± 1.2

## Data Availability

The data and analysis in this study are available on request from the corresponding author.
